# Investigation of the degradation of pitch-based carbon fibers properties upon insufficient or excess thermal treatment

**DOI:** 10.1038/s41598-017-05192-5

**Published:** 2017-07-05

**Authors:** Tae Hwan Lim, Sang Young Yeo

**Affiliations:** Technical Textile and Materials R&D Group, Korea Institute of Industrial Technology, 143 Hanggaulro, Sangnok-gu, Ansan-si, Gyeonggi-do 15588 Republic of Korea

## Abstract

To overcome the disadvantages of discontinuous conventional batch extruders, a continuous screw extruder is introduced to manufacture pitch-based carbon fibers. For a carbon fiber preparation process, an oxidation time of 8 h was determined to be optimal for obtaining desirable mechanical properties of the fibers acquiring employing the screw extruder. It is hypothesized that the differences in the properties of the carbon fibers fabricated utilizing the batch and screw extruders originate from the melt spinning time; therefore, a combined equation for the total amount of heat treatment from the pitch precursor through the oxidation process is established in this study. The crystallinity of the carbon fibers is confirmed to correspond to the differences in mechanical properties as the oxidation time increases. The poor mechanical properties of the carbon fibers that are insufficiently oxidized are a result of irregular oxidation from the sheath to the core of the fiber cross section. However, the over-oxidized carbon fibers also show poor mechanical properties than the optimal fibers. This result further affirms that excessive oxidation times cause unstable chemical bonding, which interrupts the formation of stable crystal structures after carbonization.

## Introduction

Carbon fibers attract considerable attention because they provide a high tensile strength and modulus with low weight. In particular, pitch-based carbon fibers have the inherent benefits of a lower-cost precursor and higher carbonization yield compared to using other carbon fiber precursors. The pitches derived from coal tar or petroleum through thermal treatment are classified as isotropic pitch (IP) for general-purpose carbon fibers and mesophase pitch (MP) for high-performance carbon fibers according to the mechanical properties. Notably, anisotropic MP-based carbon fibers have emerged as a variety used in higher value-added industries, such as aerospace and expensive sporting goods^1–4^. Many studies have been conducted regarding pitch polymerization to convert the petrochemical residue to the melt spinning precursor^5–8^; however, the melt spinning process at a large scale has rarely been investigated. With this technique, it is very difficult to fully purge the nitrogen atmosphere or control the rheological properties and the temperature accuracy of the molten pitch. Additionally, it is difficult to wind the as-spun pitch due to its brittleness and very low tensile strength (less than 10^−4^ GPa, *as confirmed by our experiments*).

The majority of research groups investigating pitch-based carbon fibers prepare the pitch fibers using a batch extruder composed of a cylindrical apparatus. The molten pitch is pulled out by the nitrogen extrusion pressure^9–12^. It is an easy way to obtain the as-spun pitch; however, the three main limitations of limited melting capacity, poor temperature uniformity of the molten pitch and irregular thermal treatment time according to the spinning sequence limit its use to the laboratory scale. To overcome these disadvantages, a screw extruder is introduced in our study. The continuous processable screw extruder helps to obtain uniform quality in the molten materials. In addition, controlling the screw velocity enables mass production and nozzle pressure variations, which control the mechanical properties of the fibers.

Based on previous efforts^13, 14^, we designed a single-screw extruder with a gear pump system to prepare high-density pitch fibers (The schematic image of this system is shown in Figure S1). The faster process of the screw extruder indicates that the transformed pitch fibers experience a lower thermal energy, whereas the conventional batch extruder requires sufficient time to soften the pitch for successful melt spinning. The as-spun pitch is transformed into carbon fibers through oxidation and carbonization processes, which are important for controlling the mechanical properties; therefore, many reports that provide mechanical results adapt different oxidation and carbonization conditions^15, 16^. This study focused on investigating two points: (1) the differences in properties between carbon fibers obtained using batch and screw extruders, and (2) the specific reasons why the differences occurred with respect to the total amount of thermal treatment.

As stated above, the pitch fibers prepared using batch and screw extruders are created by absorbing different amounts of thermal energy. Our study considers the total amounts of heat absorbed by the pitch during not only the oxidation and carbonization processes but also the melt spinning process to explain the characteristics of the screw extruder. In this work, four different as-spun pitches, called pitch fibers (PFs), were prepared: IP and MP fibers using both batch and screw extruders. Namely, batch-type IP (bIP PF), batch-type MP (bMP PF), screw-type IP (sIP PF), and screw-type MP (sMP PF), were melt spun to manufacture carbon fibers. The PFs were then subjected to various oxidation times of 1, 4, 8, and 12 h to verify the effects of different amounts of heat absorption. After carbonization under the same conditions, the mechanical and electrical properties of the carbon fibers were analyzed, and their crystallinities were also measured using advanced techniques^17–20^. In particular, this study attempts to interpret the crystallinity differences in terms of the various types and strengths of the chemical bonds after oxidation.

## Results and Discussion

### Optimum oxidation condition

**Figure 1:**
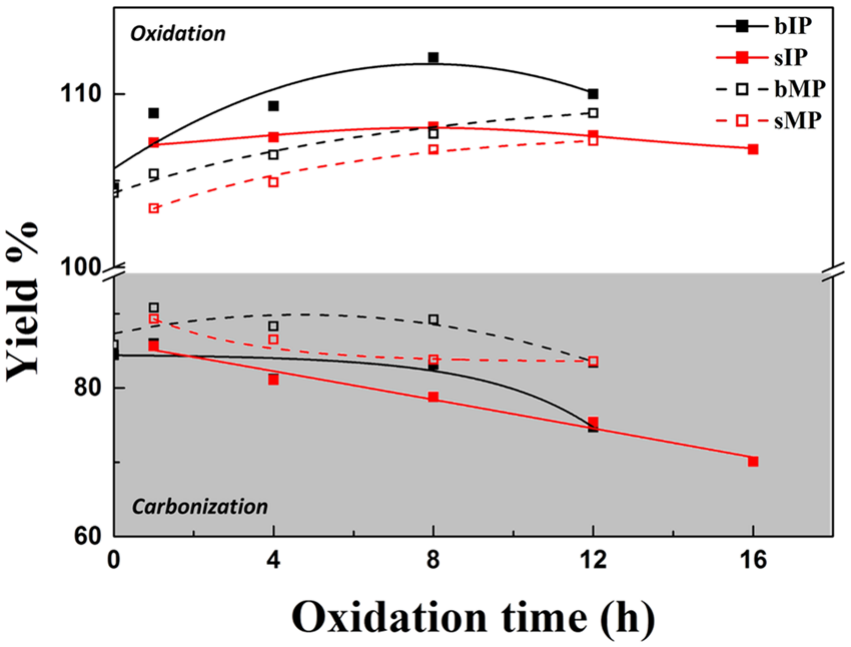
The yields of IP-based (filled squares and solid line) and MP-based (empty squares and dashed line) pitch fibers after oxidation and carbonization, prepared by batch (black)- and screw (red)-type extruder techniques.

Various carbon fibers were prepared using different melt spinning methods, pitch types and thermal treatment times through oxidation and carbonization processes. Figure 1 shows the yields for all the oxidized fibers (OFs) and carbon fibers (CFs). Those prepared utilizing a conventional laboratory-scale batch apparatus, named batch extruder (abbreviated bOF and bCF, black figures), exhibited slightly higher yields than those manufactured employing the screw extruder (abbreviated sOF and sCF, red figures). It is hypothesized that the difference is caused by the melt spinning times. With the batch extruder, a sufficient holding period at high temperature is required to eliminate impurities and low-molecular-weight pitch for the melt spinning of PFs. In contrast, the PFs prepared using the screw extruder (sPF) are transformed during a very short heating period to remove impurities. It is observed that elements other than carbon are removed during the oxidation process. The carbon and oxygen content of the OFs supports the above-stated view. Ideally, OFs should contain only carbon and oxygen because the properties of fibers can be adulterated by other atoms after carbonization. Although the yields of the sOFs were lower, it was confirmed that they contain higher percentages of C and O than the bOFs. In addition, it was verified that CFs have analogous atom content regardless of extruder type (Shown in Table S1). This result indicates that the screw extruder is feasible for fabricating CFs compared to the conventional batch extruder.

Figure 1 also shows the trends of the yields according to the oxidation times. Higher yields of OFs were observed with longer oxidation times; however, a threshold was observed after 8 h of oxidation time in the case of IP. The oxygen and carbon content of the OFs is also demonstrated to have a critical point similar to the yield tendency. The results support the theory that the oxidation sites of PF that enable bonding between carbon and oxygen are limited and that the sites differ according to the carbon layer direction, as indicated by the yield and EA results for the IP OFs and MP OFs. A compact and more-ordered carbon layer structure interrupts oxygen bonding with carbon layers^21^, which is observed in these results. Both OFs exhibit a threshold point of oxygen content; however, it is confirmed that the less-ordered isotropic structure has a greater capacity for capturing oxygen than the mesophase structure does.

The elemental content after carbonization reflects the removal of oxygen and hydrogen, and the carbon content increases through the high-temperature treatment. The changes in the carbon and oxygen content are inversely related. The oxygen content of the CFs decreases until an oxidation time of 4 h for bCFs and an oxidation time of 8 h for sCFs; however, they rebound during further oxidation. These results suggest that various oxygen bonds with carbon are formed during oxidation times of 1 to 12 h that have different bond strengths, which influence the oxygen content of both CFs after the carbonization process.

### Properties of carbon fibers

**Figure 2:**
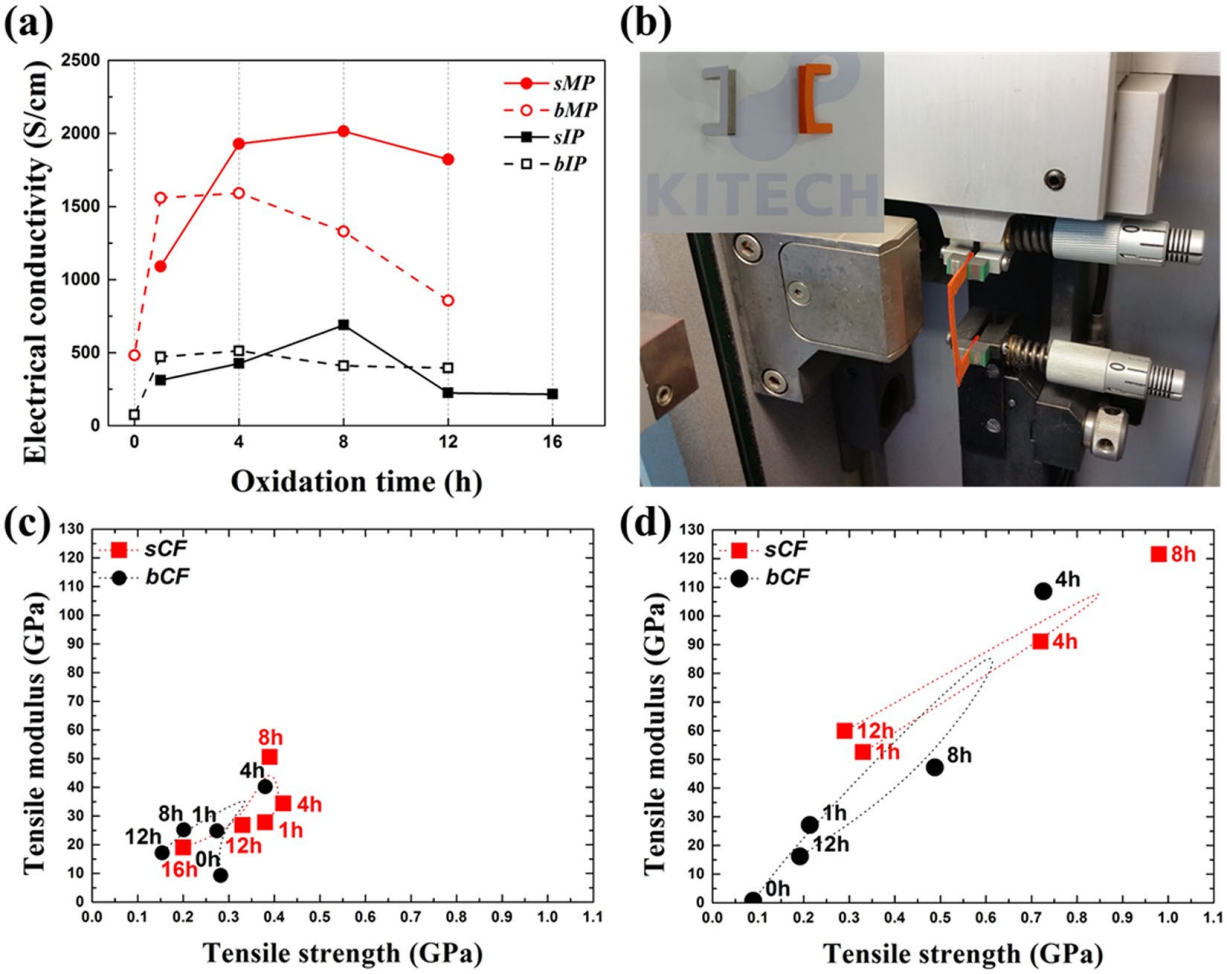
Electrical conductivity of CFs (**a**) and the device for measuring their tensile properties, with an inset image displaying the paper jig (**b**). The mechanical properties of IP-based (**c**) and MP-based (**d**) CFs.

The IP- and MP-based PFs prepared using the batch and screw extruders were transformed into CFs through oxidation and carbonization processes. The oxidation is required to ensure their infusibility after the subsequent carbonization^22^. The process enables oxygen-containing functional groups, such as hydroxyl, carboxyl, ether and ketone groups, to be attached to the surface. Different series of CFs originating from IP and MP were prepared to compare the electrical and mechanical properties of bCFs and sCFs with different oxidation times. Based on the elemental results, it was speculated that the oxygen contents significantly affected these properties of the CFs. Correspondingly, the electrical property curves shown in Fig. 2(a) were similar to the carbon content tendencies. In addition, the maximum critical points of the electrical conductivities of all CFs coincided with the lowest oxygen content of each CF. The tendencies were also similar to the mechanical properties of all CFs shown in Fig. 2(c) and (d). Naturally, the highly oriented and ordered MP CFs exhibited higher electrical and physical properties than the IP CFs did. In the case of MP CFs, 2,014 S/cm for sMP CF and 1,591 S/cm for bMP CF were the maximum values measured for 8 h (sMP8 CF, the number following the type of pitch references the oxidation time) and 4 h oxidation times (bMP4 CF). Additionally, 689 S/cm for sIP8 CF and 513 S/cm for bIP4 CF were the highest electrical properties. Note that the tensile strength of IP CFs did not considerably change with oxidation time, at approximately 0.2~0.4 GPa, whereas that of MP CFs increased from 0.2 to 1.0 GPa for the different oxidation times. The tensile modulus of both CFs gradually increased to 53 GPa for sIP8 CF and 125 GPa for sMP8 CF and then decreased beyond the critical oxidation time.

When these results were combined, without regard to pitch type, oxidation times of 8 h and 4 h resulted in the maximum properties for sCF and bCF, respectively. In addition, it was confirmed that sCFs show higher properties than bCFs as a result of the pressure difference during the melt-spinning process. It was previously reported that a higher pressure at a final outlet, such as a spinning nozzle, generates an ordered fiber alignment^23^. For this reason, it is beneficial to use a screw extruder to easily control and enhance the nozzle pressure. In addition, it was confirmed that the optimum properties of CFs are obtained at different oxidation times: 8 h for sCFs and of 4 h for bCFs. It is speculated that these results are caused by the melt-spinning process period. As mentioned above, a considerable amount of time, over 2 h, is required to obtain a successful melt-spinning process in the batch extruder (The specific procedure is shown in Table S2).

**Figure 3:**
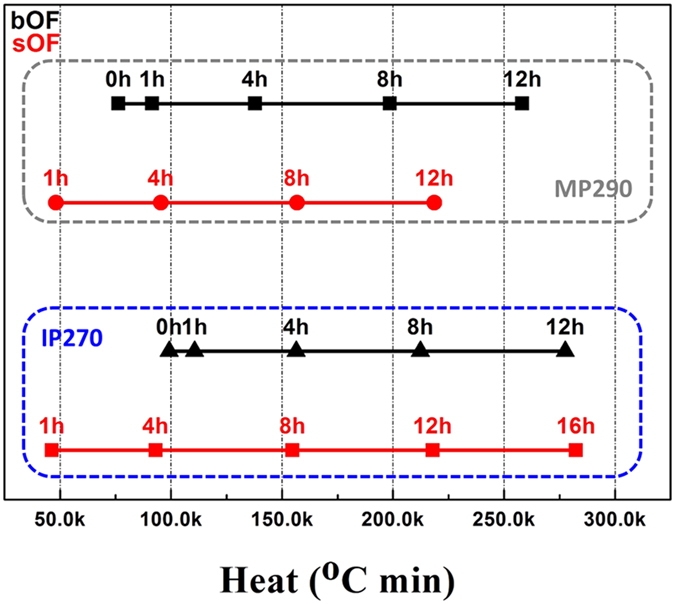
Heat absorption calculated by equation (1) for the experimental OFs. The red and black lines represent sOFs and bOFs, respectively.

Separate and independent studies on the differences in properties with respect to the melt-spinning temperature and oxidation and carbonization conditions have been previously performed^24, 25^. We attempted to prepare and compare the properties of CFs using different processing methods; therefore, combination tools for temperature and heating times are introduced to interpret the properties of the CFs in this study. To consider heat absorption, we determined the sum of heat absorption by integrating the temperature with time from the melt spinning to the oxidation using equation (1) in the Methods section. Figure 3 presents the calculated results for both IP and MP OFs fabricated using screw and batch extruders. The results shows that the sOFs and bOFs yielded from their respective optimum oxidation times of 8 h and 4 h exhibit similar heat absorptions of approximately 150.0k °C min. It was also confirmed that the CFs with over 200.0 k °C min of heat absorption have low mechanical properties. The index and corresponding properties strongly suggest that there must be a suitable heat absorption to obtain pitch-based CFs with optimum properties regardless of pitch type and fiber preparation method.

**Figure 4:**
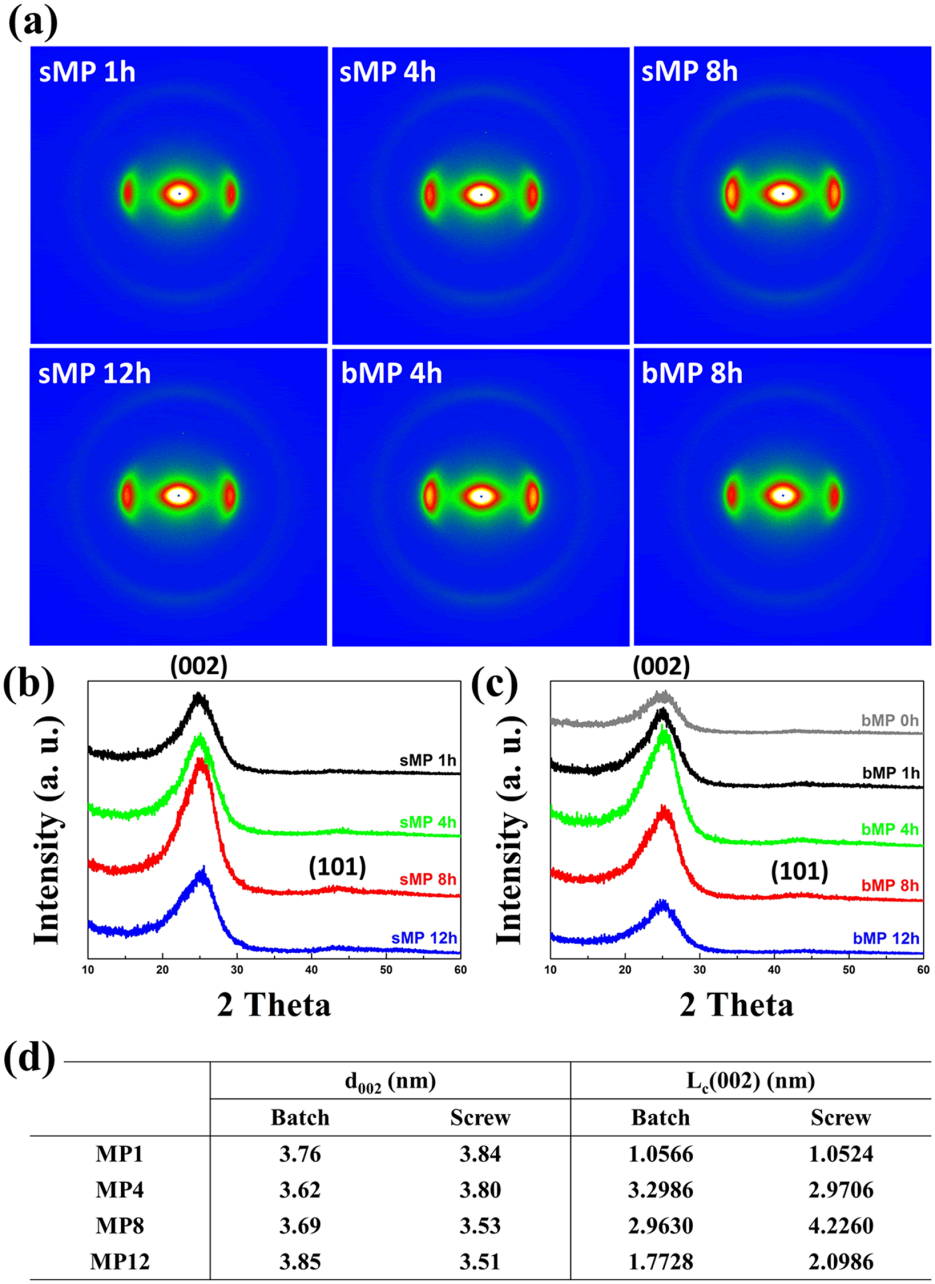
(**a**) 2D SAXS patterns of MP CFs. (**b**) and (**c**) show the XRD profiles of sMP and bMP CFs according to their oxidation time. Table (**d**) displays the XRD parameters for the MP CFs.

The decrease in properties with excess heat absorption is explained by the crystallinity, which is clearly shown in the two-dimensional small-angle X-ray scattering (2D SAXS) images and diffraction patterns in Fig. 4(a). sMP and bMP CFs show differences in sharpness and 2θ angle position according to oxidation time, Fig. 4(b) and (c). Consistent with the mechanical and electrical properties, the (002) peak of the sMP8 CFs and rMP4 CFs is sharper and at a higher 2θ angle than that of the CFs manufactured using other oxidation times. This result indicates that the interlayer spacing decreased and that larger crystallites formed. sMP12 CF and rMP8 CF presented a broader (002) peak than each optimum CF, consistent with our expectations based on the mechanical property results. The interlayer spacing (d_002_) and crystallite stacking height (L_c (002)_) calculated based on the parameters (Fig. 4d) help in supporting the X-ray diffractometer (XRD) results. The interlayer spacing slightly decreased with increased oxidation times as indicated by the 2θ shift to higher angles, and it indicated a lower threshold point.

The crystal size derived from the full width at half maximum (FWHM) data is the key point corroborating the decrease in the properties of the CFs according to the amount of overheating (The IP CFs also have the same tendencies; however, because of their lower crystallization characteristics, it is more difficult to discriminate the crystallinity differences than in the MP CF series. The patterns and parameters of the IP CFs are shown in Figure S2). The optimum CFs, namely, sIP8, sMP8, bIP4, and bMP4, form the largest crystal domains. It is observed that the higher crystal heights provide a more compact structure; therefore, the CFs with a large L_c_ (002) show higher mechanical properties derived from intermolecular forces, such as van der Waals forces^26, 27^. Another peak, (101), reveals the existence of three-dimensional graphitic crystallinity and radial texture on the cross section of the carbon fiber. The higher turbostratic structures in the cross section of these optimum CFs are also demonstrated by the (101) peak arising from the growing and orderly graphene-layer stacking crystallites.

**Figure 5:**
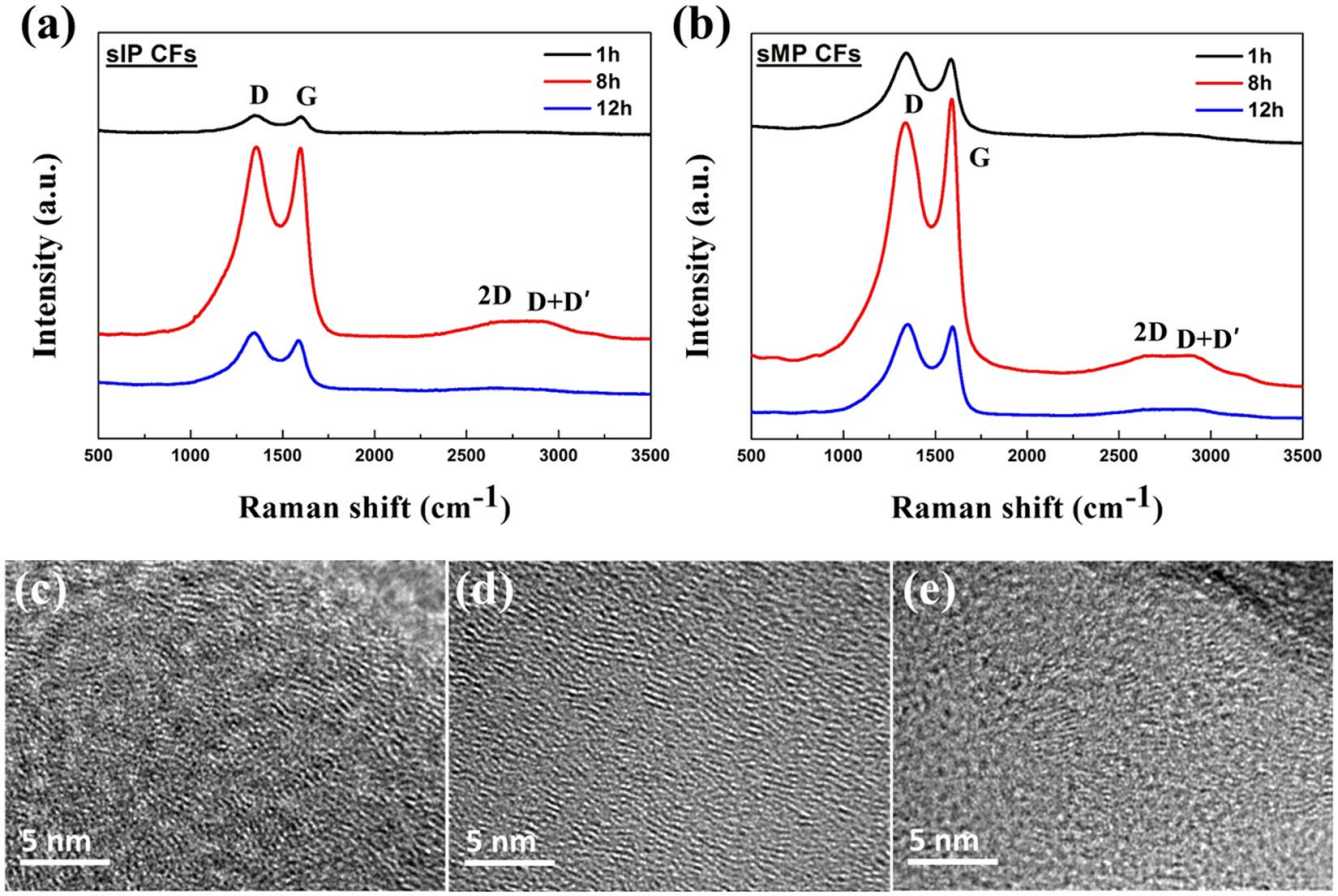
Raman spectroscopy of (**a**) sIP CFs and (**b**) sMP CFs. The stacked crystallite heights and inter-spacing layer information for (**c**) sMP4 CF, (**d**) sMP8 CF, and (**e**) sMP12 CF were supported by TEM images.

From this point forward, the further studies investigating the reasons behind the mechanical property decrease from overheating are focused on sCFs because (1) bCFs and sCFs show a similar tendency for property changes and (2) sCFs are in accordance with our research objective of enabling a continuous process. The Raman spectra and graphical images in Fig. 5 reflect the structural characteristics of graphitic carbon materials. The Raman spectra in Fig. 5(a) and (b) show smaller FWHMs of the G and D bands, which indicate higher purities of the disordered and ordered graphite structures. It is widely known that the G band at 1580 cm^−1^ indicates an ordered graphite crystallite structure attributed to the in-plane tangential stretching of the sp^2^ C=C bond, whereas the D band at 1360 cm^−1^ reflects the disordered graphite structure due to sp^3^ C-C bonds. In addition, the 2D and D + D′ bands at 2680 cm^−1^ and 2940 cm^−1^ are the overtone and combination of the D band due to two phonons with opposite momenta, respectively^28, 29^. The ratio of the integrated intensities of the G and D bands (A_G_/A_D_) represents the degree of graphitization. The Raman spectra and the A_G_/A_D_ trends of the sIP and sMP CFs also endorse the previous property and crystallinity results. The sharp G band of sMP8 CF indicates that it has a higher purity of ordered graphite structures than the other sMP CFs, and the degree of disorder is increased by excessive oxidation times over 8 h.

The stacked crystallite height and crystallinity of the sMP CFs as determined by the XRD parameters are visually shown in the transmission electron microscopy (TEM) images in Fig. 5(c)–(e). sMP8 CF exhibits the most well-aligned graphite structure, and the over-heat-treated sMP12 CF exhibits the least crystalline structure, which aligns with the Raman and XRD results. Although it is difficult to accurately identify crystallite information, it is possible to understand the approximate stacked height and crystallinity of the sMP CFs. Overall, the results indicated that the overheating process had a negative effect on the creation of large crystallites in CFs by destroying the initial stacking structure. These results demonstrate that the sMP12 CF exhibits debased mechanical properties due to the lower intermolecular forces between the graphene layers.

### Causes of the property shifts with different oxidation times

Oxygen content differences according to oxidation times reveal that the liquid crystal-like mesophase PF surface is not only difficult to oxidize but also retards the diffusion of oxygen into the fiber cores because of its rigid structure compared to that of amorphous isotropic PF. Insufficient oxidation creates fusibility during the subsequent carbonization; therefore, it reduces mechanical strength and other characteristics^30^. Our mechanical results (Fig. 2c and d) support this explanation. The property difference in the IP CFs is relatively lower than that in the MP CFs because of the oxygen bonding constituents varying with pitch properties, and it suggests that sufficient oxidation times are required to obtain the highest properties.

**Figure 6:**
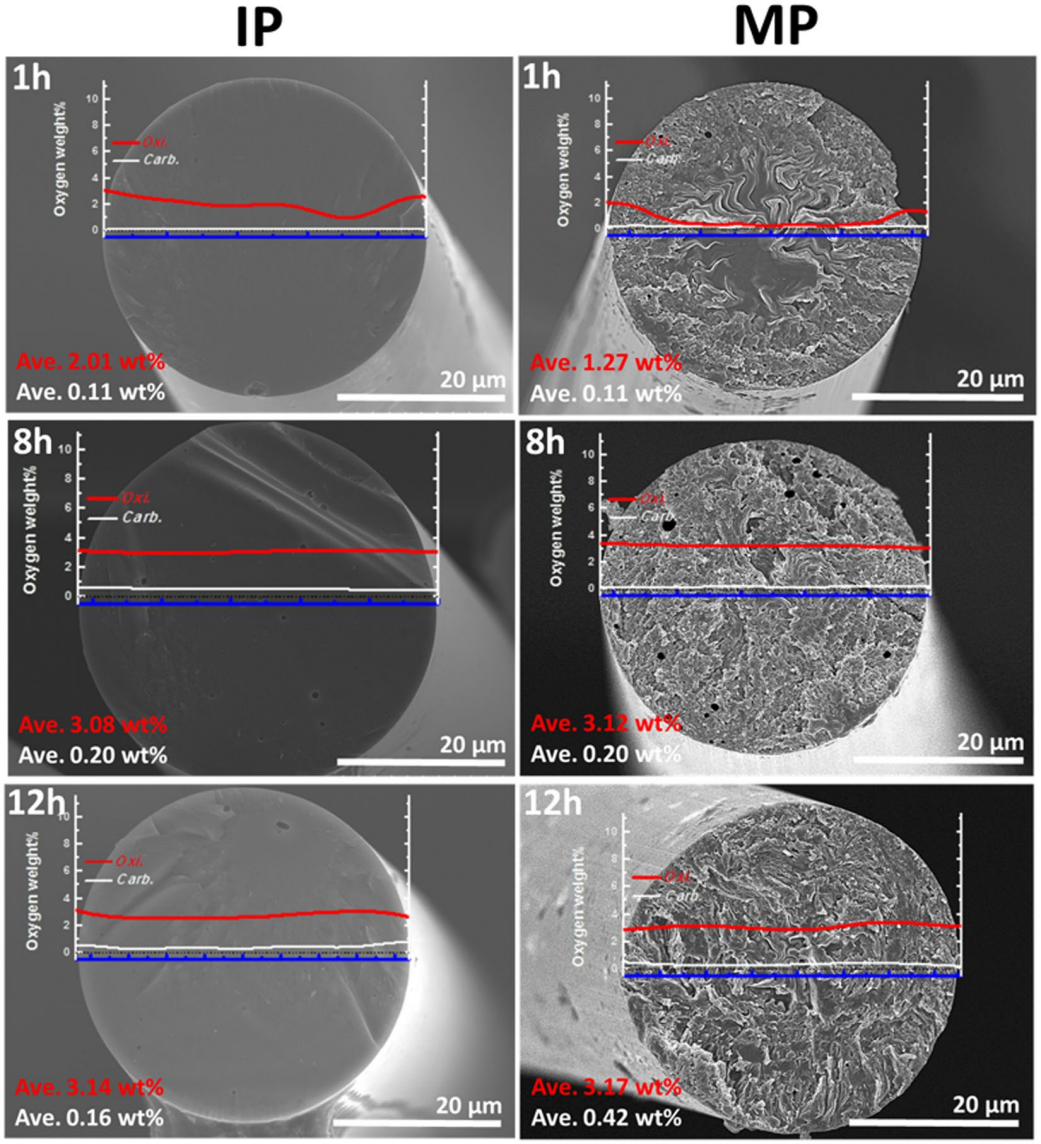
Cross-section SEM images of CFs prepared with different methods and oxidation times. The oxygen distributions across the fiber diameter of each OF and CF are overlapped on the SEM images. The red and white lines indicate the values after oxidation and carbonization, respectively.

This rationale is supported by the oxidation regularity of the OFs; these results are shown in Fig. 6 combined with the electron probe micro-analysis (EPMA) line profiles of the OFs and CFs with cross-sectional SEM images of the CFs. As shown by the line profile results, the oxygen content after the insufficient oxidation time of 1 h shows irregularities between the sheath and core. The heterogeneous morphology observed in the cross-sectional image of MP1 CF provides visual support for the oxidation results (the EPMA result of IP1 OF also supports the oxidation time being insufficient; however, it is impossible to confirm based on the image due to isotropic characteristics). The MP CFs prepared using our technique show a random texture, and the morphologies are the products of highly stacked graphite layers. The SEM image of the core of an MP1 CF shows a plain cross-sectional surface due to fusion during the carbonization process. The melted parts of the CF interrupt the creation of a highly crystalline structure; therefore, the graphene stacking heights of sIP1 and sMP1 CFs are very low compared to those of sIP8 and sMP8 CFs, as shown in Figs 4(d) and S2(d) ^31^. The sufficiently oxidized samples, IP8, IP12, MP8, and MP12 CFs, exhibit regular oxygen content and homogeneous textures irrespective of the sheath-core regions. Combined with the crystallinity results, sufficient oxidation times that facilitate regular oxygen content from the sheath to core are required to fabricate high-performance CFs.

The reason why excessive heating times, with sMP12 and sIP12 CFs as representative samples, induce a degradation of the properties should be examined. Previous studies have reported that various types of bonds between carbon and oxygen, such as carbonyl (C=O), carboxyl (-COOH) and ether bonds (-COC-), are created in amorphous regions rather than at the stable surface composed of packed aromatic structures^32^. We predict that different types of carbon-oxygen bonds are formed at different ratios for various oxidation times, and it is expected that these bonds, which have different strengths, play important roles in the mechanical properties of CFs after carbonization. Therefore, X-ray photoelectron spectroscopy (XPS) analyses of the IP and MP OFs were used to identify the types of bonds according to the oxidation times. The oxygen contents measured by XPS (Table S3) also indicates that the EA results and content differences between the IP and MP OFs are due to their distinct chemical structures. For these reasons, the functional groups related to the carbon and oxygen of IP OFs are more clearly distinguished than those of MP OFs.

**Figure 7:**
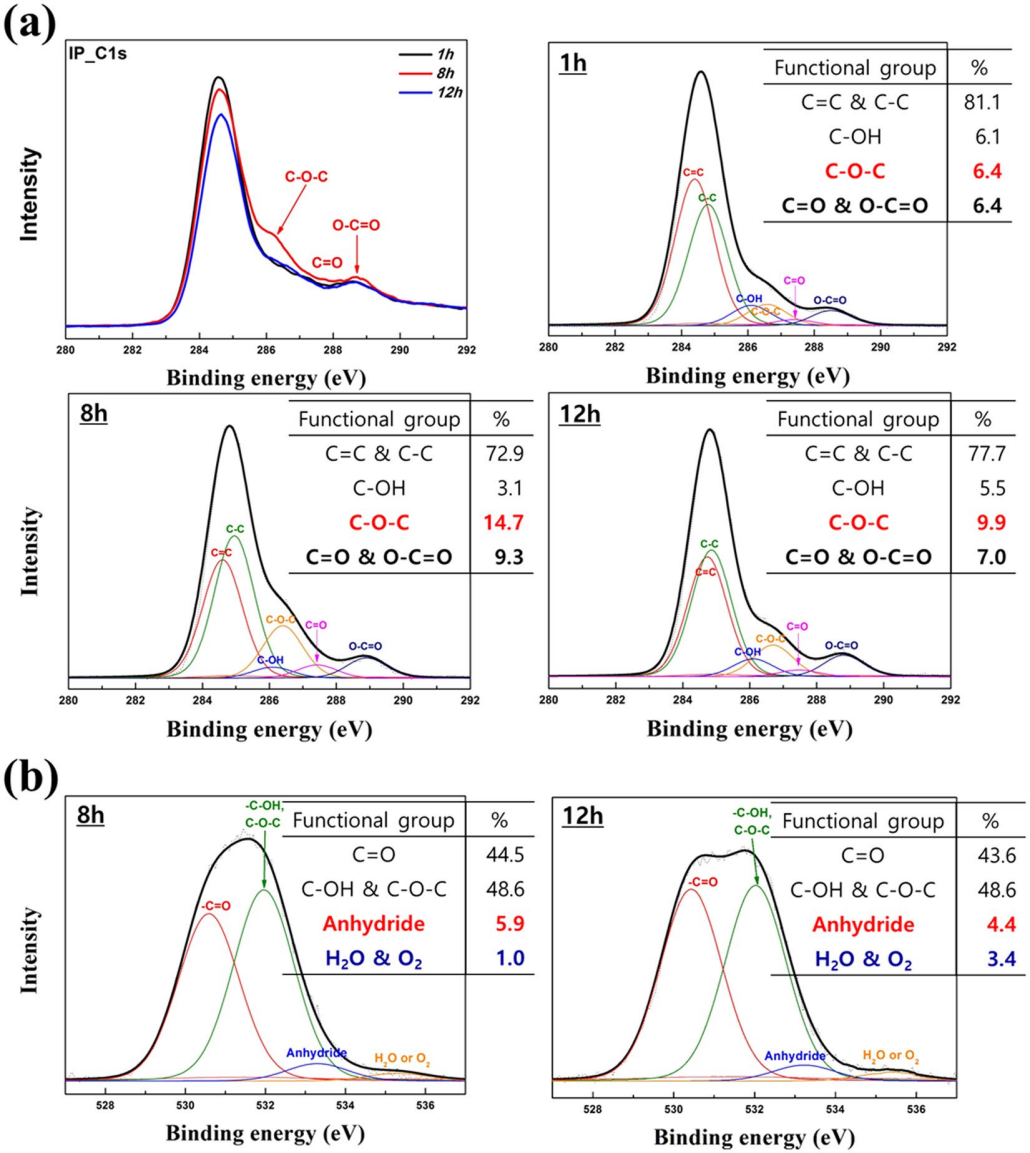
X-ray photoelectron spectra of IP OFs prepared with different oxidation times: (**a**) C1s and (**b**) O1s peaks.

Figure 7 presents the C1s and O1s XPS spectra and the molar ratio distributions of the IP OFs. All curves are fit using Gaussian and Lorentzian equations. The C1s peaks of all the OFs are deconvoluted into six peaks centered at 284.6, 284.9, 286.1, 286.6, 287.4, and 288.6 eV, which are attributed to the graphitic C=C, aliphatic C-C, C-OH, -C-O-C-, C=O, and O-C=O bonds, respectively. As shown in Fig. 7(a), the levels of carbon-oxygen bonds are increased, compensating for the decrease in C-C and C=C bonds. IP8 OFs have more ether and C=O bonds than the other IP OFs do. Previous studies have demonstrated that ether groups help to enhance the flexibility of polymer chains^33–35^. Although most of the oxygen bonds are eliminated after the carbonization process, some of the remaining strong C-O-C bonds appear to have a beneficial effect on the higher modulus of the CFs. Oxygen bonded with the carbon of the OFs acts as a shield to protect the infusibility through carbonization to form larger crystallites. However, it was already demonstrated in Fig. 2(c) that sIP12 CF exhibits considerably lower mechanical properties than sIP8 CF even though their oxygen content is almost identical. We determined that the reason why the mechanical properties are different is the existence of bonding between oxygens, as verified by the deconvoluted O1s peaks in Fig. 7(b). The peak centered at 535.2 eV represents water or O=O bonds, and it indicates that O_2_ molecules are simply laying on the carbon surface and are not chemically bound^36–38^. The results suggest that proper oxidation times create strong and necessary C-O bonds, whereas the overheating process used with the sIP12 OF generates unnecessary bonding between oxygens, diminishing the significant C-O bonding.

**Figure 8:**
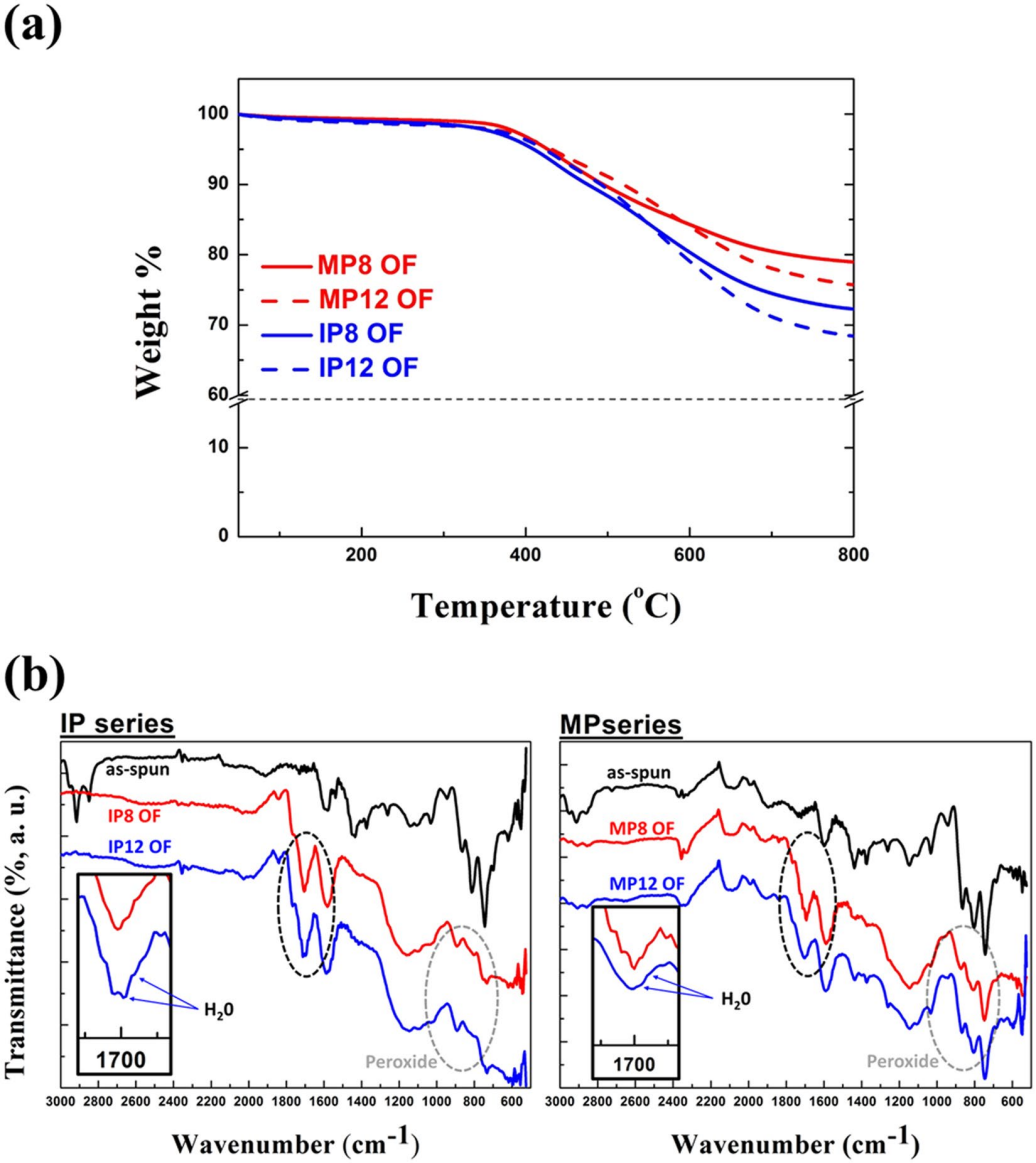
(**a**) TGA curves of different OFs under N_2_ gas to compare the weight remaining after oxidation durations of 8 h and 12 h and (**b**) FT-IR spectra of IP and MP OFs.

It is determined that the O=O bond is lying on a site with weak thermal stability; therefore, the bonding site acts as a defect to destroy the crystal structure after carbonization. Due to the low oxygen content, the differences in the molar ratio distributions of the sMP OFs are relatively lower than those of the sIP OFs (Figure S3). However, they reveal tendencies analogous to the analyzed results of the sIP OFs. Evidence for the weak thermal stability of the bonding between the carbon and oxygen molecules is provided by the thermal gravimetric results shown in Fig. 8(a). Assuming that the thermogravimetric process simulates the carbonization process, sIP12 and sMP12 OFs show greater degradation behavior in N_2_ gas than the sIP8 and sMP8 OFs so, even though they contain similar oxygen content. In addition, Fourier transform infrared spectroscopy (FT-IR) results (Fig. 8b) strengthen our assertion from the XPS results. OFs prepared by two different pitches, IP and MP, showed similar tendency between appropriate and excess oxidation times, and it was confirmed that the over-oxidized OFs had stronger peaks at 1680 and 1630 cm^−1^, which indicate the bending vibration of water^39, 40^. Although the difference was not significant, weak -O-O- bonds of OF 12 h including peroxide group were also slightly greater than those of OF 8 h. The FT-IR results support the idea that the over-oxidized OFs have more oxygen atoms; however, the over-oxidized OFs exhibit lower mechanical properties because they retain more weak oxygen bonds thermally.

In summary, insufficient oxidation times are demonstrated to have an influence on the irregular oxidation from the sheath to the core, and excessive oxidation times lead to ephemeral chemical bonding that weakens the sturdy crystal structure.

## Conclusions

Pitch-based carbon fibers are prepared employing a screw extruder for a continuous process. Two different pitch precursors, IP and MP, were successfully formed into fibers by melt-spinning, and the continuous screw extruder was used to replace the conventional batch extruder. The final materials were manufactured through thermal treatments, including oxidation and carbonization processes. In this work, different oxidation times were applied to elucidate the thermal effect on the mechanical properties. According to the results, the optimum oxidation times are 8 h for sCFs and 4 h for bCFs. In addition, the total heat absorption integrated temperatures with time from the melt-spinning through the oxidation process to generate the optimum properties were confirmed to be almost identical regardless of the type of melt spinning. XRD, 2D SAXS, Raman and TEM analyses explained differences in properties based on the crystallinity of the CFs. These analyses illustrated that the longer oxidation times up to 8 h led to higher crystallinity due to higher graphite stacking heights. However, the over-heat-treated sIP12 and sMP12 CFs exhibited crystallinity degradation. We focused on investigating the causes for this in this study; therefore, two major analyses were conducted: confirming the oxidation regularity by EPMA line profiles and examining the differences in types of chemical bonds by XPS. Consequently, it was determined that insufficient oxidation times negatively influence the oxidation regularity from the sheath to the core, which causes low mechanical properties. Furthermore, excess oxidation causes unstable O=O bonding rather than stable C-O and C=O bonds, which helps to protect the fusibility; therefore, it interrupts the compact crystal structure after carbonization. The experimental results may provide helpful guidelines for mass production, and the discussions have great significance for clarifying the reason behind these optimum times exist based on chemical bonding analysis.

## Methods

### Materials

IP and MP were purchased from Anshan Sinocarb Carbon Fibers Co., Ltd. (China) and E&E Chem. (Korea), respectively. The softening points of IP and MP were measured to be 274.2 °C and 292.7 °C using a softening point determination system (DP90, Mettler Toledo, Switzerland) following the American Society for Testing and Materials (ASTM) guidelines D3104 and 3461. The values are the averages of 5 measurements. These and other basic properties of the pitches are presented in Figure S4. During thermogravimetric analysis in an air atmosphere, both the MP and IP exhibited a weight increase in the range of 200 to 400 °C as a result of the oxidation reaction. It is reported that preparing MP requires more thermal and purification processes than preparing IP^41^. Elemental analysis (EA) and inductively coupled plasma atomic emission spectrometry (ICP-AES) confirmed that more carbon and fewer impurities are present in the MP, as mentioned above.

### Pitch fiber preparation

Two different types of extruders were used to compare the product properties according to the spinning method. The first is a batch extruder formed of stainless steel with a capacity for 30 g of pitch, and pitch fiber was extruded using a nitrogen pressure of 1.0 bar. A single-hole spinneret nozzle with a diameter of Ф0.5 mm and L/D = 5.0 was used to prepare pitch fiber. Prior to spinning, the pitches in the apparatus underwent a nitrogen gas circulation process at 150 °C for 1 h to eliminate impurities and low molecular weight pitches.

The second type of extruder is a screw extruder (Brabender, Germany), which is the focus of this study. The main spinning temperatures of the barrel, gear pump, and nozzle are similar to those of batch extruder systems, at 320 °C for IP and 340 °C for MP. A multi-hole spinneret with a diameter and L/D of Ф0.5 mm and 5.0, respectively, was used. A single screw was employed, and a speed of 20 rpm was determined by considering the torque, terminal pressures, thermal behaviors and pitch outputs. A Ф300 mm winder was used to reduce curvature because the as-spun pitch is difficult to wind because of its brittleness. In addition, detachable and punched plate-based bobbins were employed to attain a continuous melt-spinning process. The PFs were wound at winding speeds of approximately 350 m/min to obtain fibers with diameters of less than 30 μm. In practice, pitch powder is difficult to uniformly supply into the screw extruder because of its agglomerative behavior at the feed throat due to heat and structural factors. To overcome this operational problem, pitch pellets were prepared using a twin-screw extruder (BA-11, Bautek, South Korea) prior to melt spinning the PFs. It was confirmed that the softening points before and after the pelleting process show little difference; moreover, this process exerts a positive effect for reducing impurities. The properties of the pitch powder and pellets are shown in Table S4.

### Carbon fiber preparation

An air-circulated box furnace with dimensions of 400(W) × 400(D) × 400(H, mm) was used to oxidize the PFs. Different oxidation times were selected to investigate the properties of the carbon fibers: 1 h, 4 h, 8 h and 12 h, which refer to the period for which the temperature was maintained. The heating rate was maintained at 1 °C/min until 280 °C, and the furnace was cooled to 100 °C within 4 h after oxidation finished. The pitch fiber was oxidized while being wound on the punched plate-based bobbin to maintain fiber tension. Our bobbin and oxidized sample images are shown in Figure S5. Carbonization was conducted under a N_2_ atmosphere at 1,000 °C with a heating rate of 5 °C/min using a tube-type furnace. Identical carbonization conditions were employed to determine the differences in properties based on the oxidation conditions in this study.

To clarify the oxidation time effect, it is believed that the melt-spinning conditions should be considered because the amounts of heat absorbed during the oxidation of fibers prepared using the batch and screw extruders are considerably different. We established an equation to estimate the amounts of heat absorbed based on the heating temperature and time during melt spinning and oxidation. The equation is shown below:

1 $$\sum {\rm{H}}({\rm{K}}\cdot \,{\rm{\min }})=\frac{{\int }_{0}^{{t}_{m}}{\Delta }Tdt+{\int }_{0}^{{t}_{f}}{\Delta }Tdt}{{m}_{f}/{m}_{i}}$$

where ∑H is the total amount of heat absorption, m_i_ and m_f_ are the initial and final masses after the oxidation process, ΔT is the change in temperature during melt spinning and oxidation, and t_m_ and t_f_ are the total periods of melt spinning and oxidation, including the heating rate, respectively. The heat absorbed during carbonization is excluded because it is the same.

### Characterizations

An FE-SEM (SU8230, Hitachi, Japan) was used to obtain images of the cross sections and fiber diameters. An elemental analyzer (EA, E1110, CE Instruments, United Kingdom) and energy-dispersive X-ray spectrometer (EDS, Hitachi, Japan) were used to determine the oxygen contents and oxygen-containing functional groups in the PFs, OFs and CFs. The electrical properties of the CFs were measured using a 2-point probe measurement system (HiTESTER 3280-20, HIOKI E.E. Corp., Japan). This measurement is suitable for determining the electrical conductivity of fiber materials. Silver paste and an insulating glass plate were applied to measure the electrical resistances of the CFs. The electrical conductivity (S/cm) was measured by the 2-point probe instrument. The mechanical properties of the CFs were determined using an automatic linear density and tensile tester for single fibers (Favimat, Textechno H. Stein GmbH & Co. KG, Germany) following JIS R 7601. The fibers are difficult to grip because of the brittleness of CFs, which induces breaks at the jaws of the tensile testing machine. Therefore, an additional paper jig was introduced to effectively measure the strength and modulus of the CFs (shown in Fig. 2b). The crystallinity and interlayer spacing were analyzed using a high-resolution XRD (HR-XRD, Rigaku, USA; Cu Kα radiation, 45 kV and 200 mA) and supported by Raman spectroscopy (Bruker, Germany, λ = 514 nm), 2D SAXS (Rigaku D/MAX-2500, USA, 50 kV and 100 mA) and Cs-corrected TEM (Cs-TEM, JEM-ARM200F, JEOL, USA). d_002_ and *L*_c (002)_ were calculated using Bragg’s formula and Scherrer’s equation, respectively^42^.

The oxygen content in the cross sections of the OFs and CFs was determined by EPMA (Shimadzu 1600, Japan; beam current: 20 nA, beam size: 1 μm). The oxygen distribution across the fiber diameter was measured using the line profile method to observe the difference in oxygen content from the surface to the core of the OFs and CFs. The types and relative contents of chemical bonds between carbon and oxygen were identified through XPS (Axis Nova, KRATOS, UK) utilizing monochromatic Al Kα radiation (1486.6 eV). Thermogravimetric analysis (TGA, Q500, TA Instruments, USA) and FT-IR (Nicolet 760, Thermo Fisher Scientific, USA) were employed to support our XPS results confirming thermal degradation and weak bond existence, respectively.

## Electronic supplementary material

Supplementary information
