# Supplementary material for: Investigation of the degradation of pitch-based carbon fibers properties upon insufficient or excess thermal treatment
Source: Sci Rep. 2017 Jul 5;7:4733. doi: 10.1038/s41598-017-05192-5 (PMC5498616; doi:10.1038/s41598-017-05192-5)
Supplement: Supplementary file 1 — Supplementary information [file 41598_2017_5192_MOESM1_ESM.pdf]

Investigation of the degradation of pitch-based carbon fibers properties upon insufficient or excess thermal treatment

Tae Hwan Lim and Sang Young Yeo*

Technical Textile and Materials R&D Group, Korea Institute of Industrial Technology, 143 Hanggaulro, Sangnok-gu, Ansan-si, Gyeonggi-do, 15588, Korea, Republic of

*miracle@kitech.re.kr

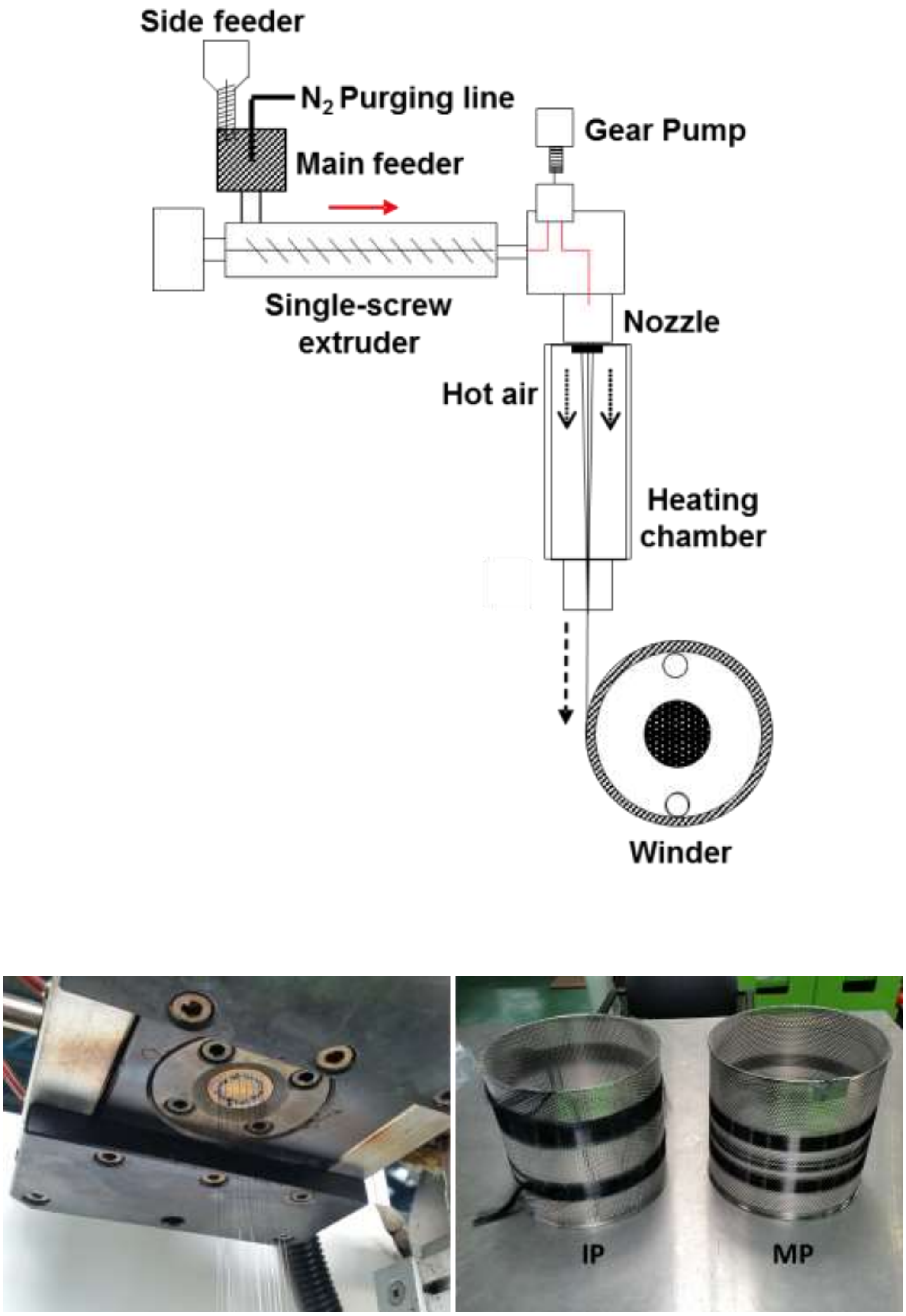

Figure S1. A schematic of the screw extruder for the continuous process and for manufacturing high-performance carbon fibers (top). The photographic images of the melt-spinning process and as-spun fibers are illustrated in the bottom images.

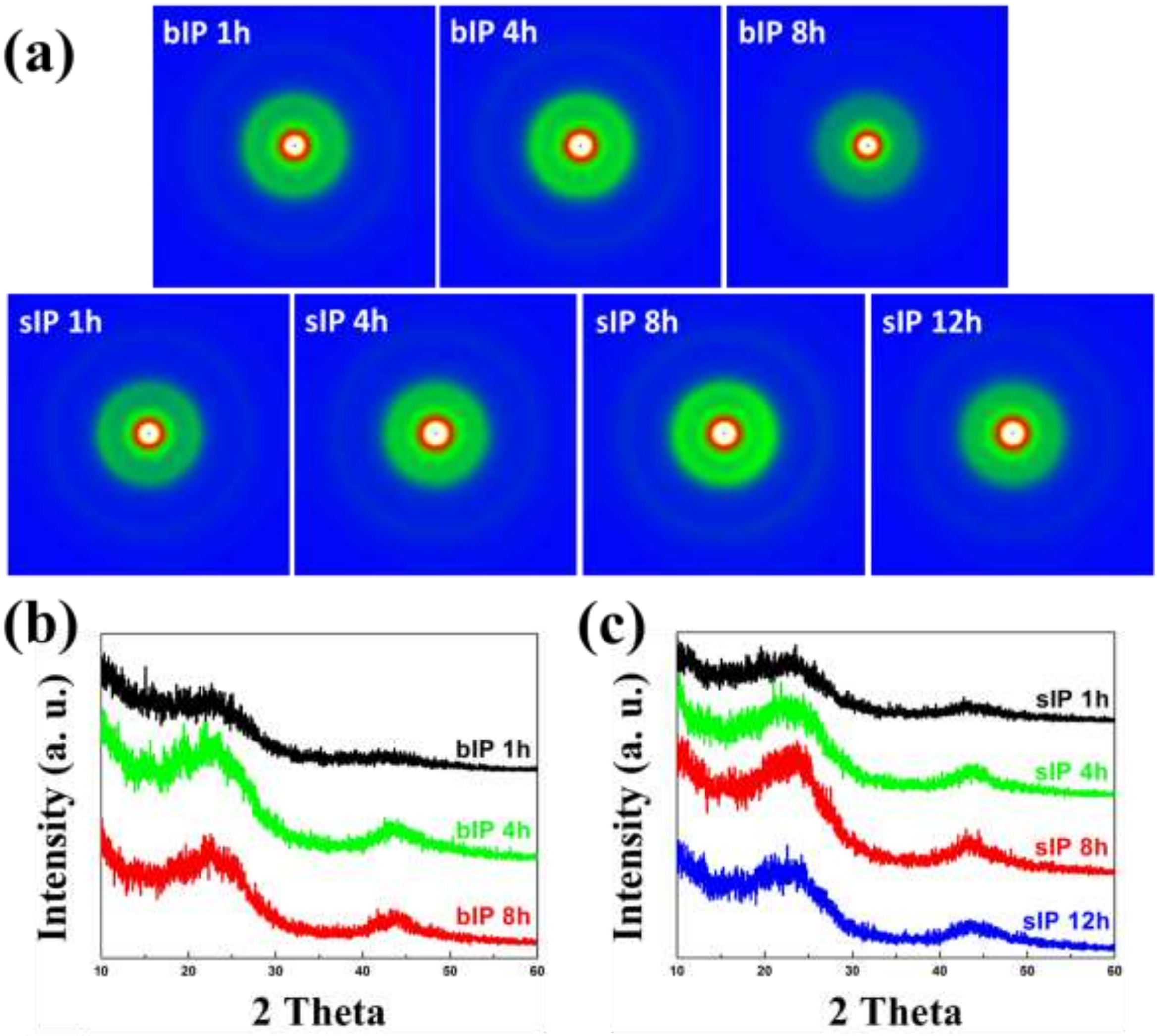

(d)

	d_{002} (nm)		$L_c(002)$ (nm)	
	Batch	Screw	Batch	Screw
IP1	3.70	3.78	1.1393	1.1023
IP4	3.57	3.77	1.9203	2.0786
IP8	3.50	3.54	1.8046	2.1133
IP12	3.68	3.53	0.2499	1.0567

Figure S2. (a) 2D SAXS patterns of IP CFs. (b) and (c) show XRD profiles of bIP and sIP CFs according to their oxidation time. Table (d) exhibits XRD parameters for MP CFs.

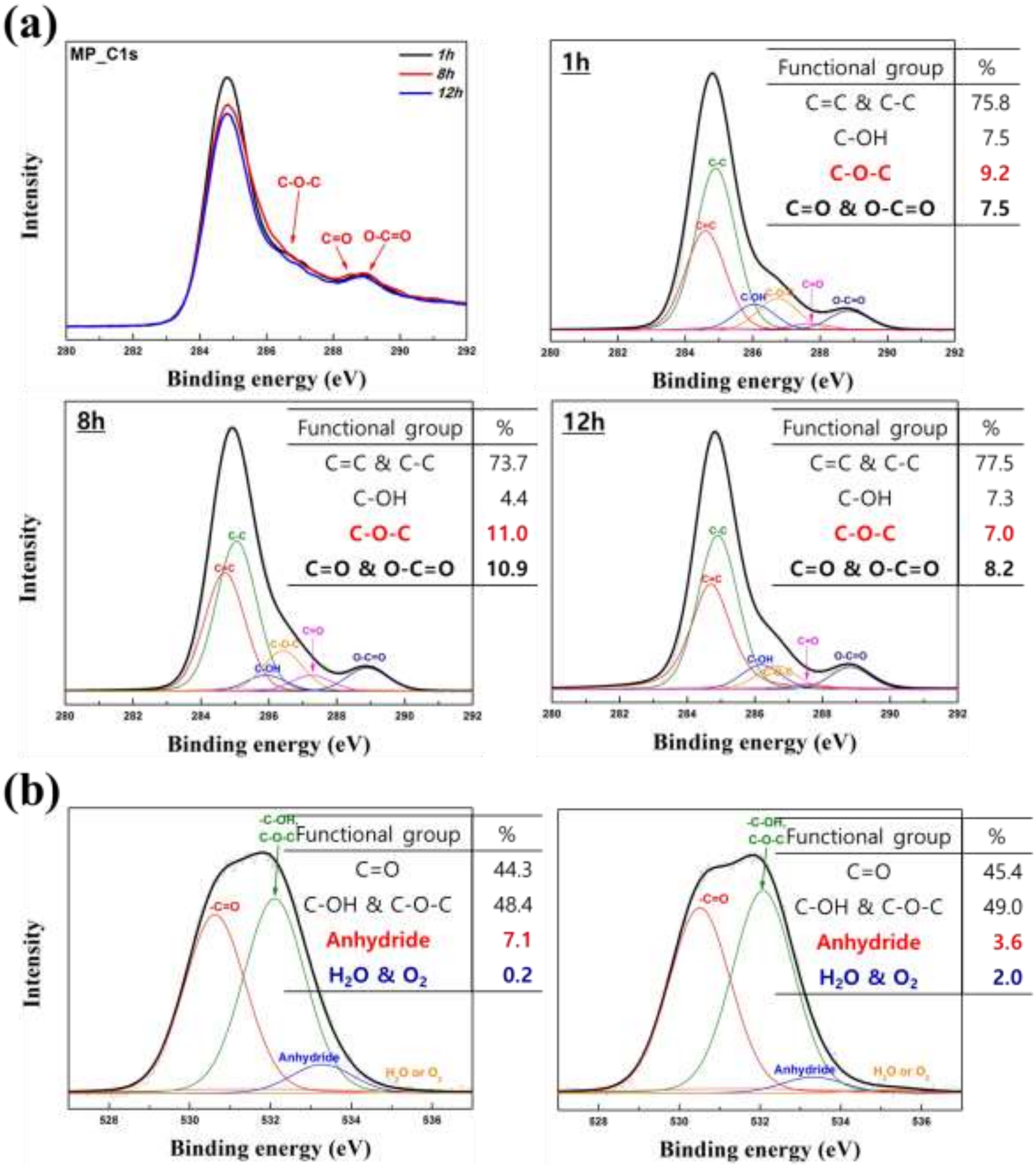

Figure S3. X-ray photoelectron spectra of MP OFs prepared with different oxidation times: (a) C1s and (b) O1s peaks.

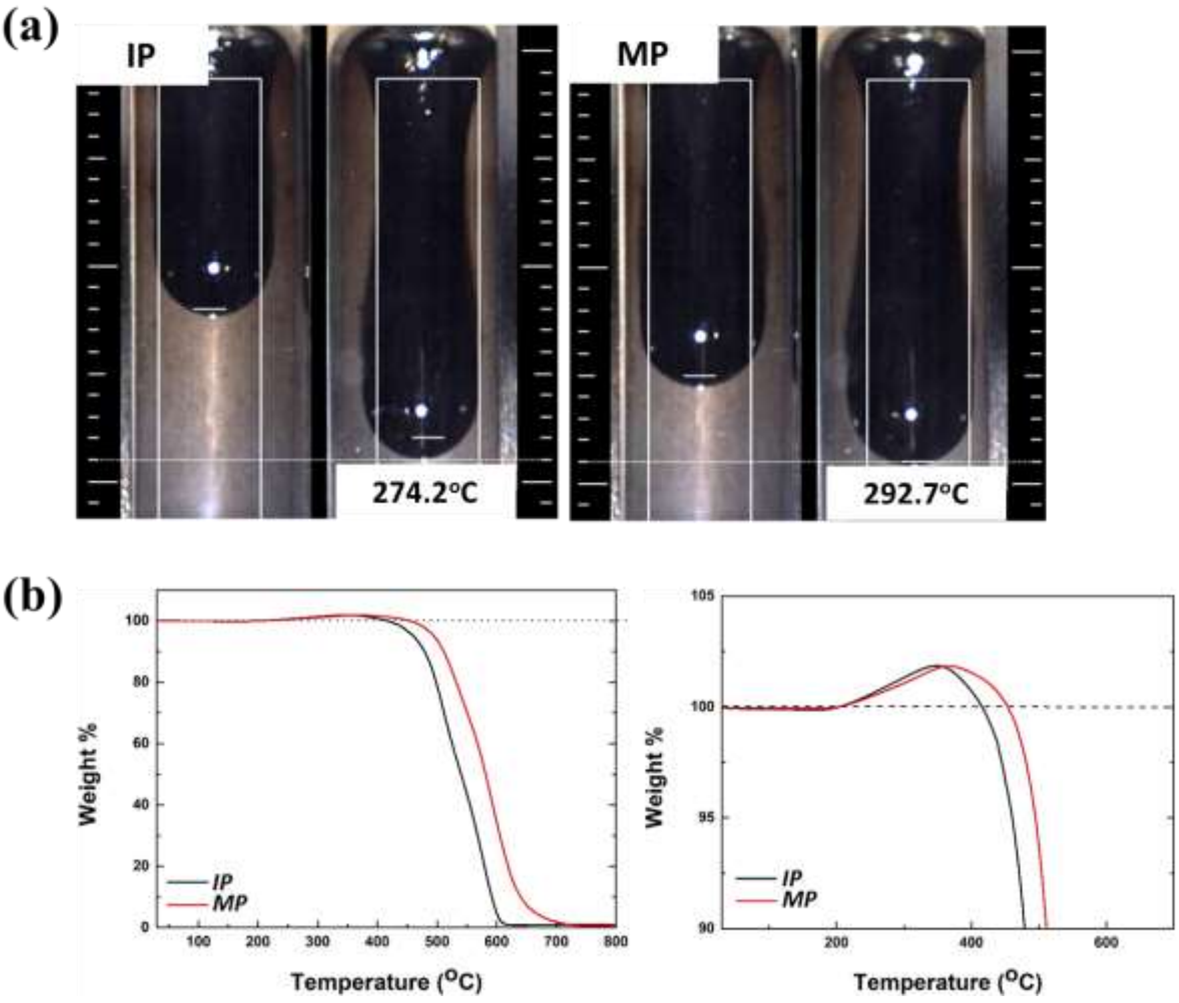

Sample	EA (%)					ICP-AES (mg/Kg)				
	C	N	H	S	O	Si	Fe	Mg	Cu	Al
IP	92.18	<0.10	5.22	<0.01	2.60	2,204	25	4	5	4
MP	94.55	<0.01	4.29	0.35	0.71	<1.0	<1.0	15	49	<1.0

Figure S4. (a) Softening point measurement images and (b) thermogravimetric curves for the oxygen gas of IP and MP. The bottom table shows the elemental components for both pitches.

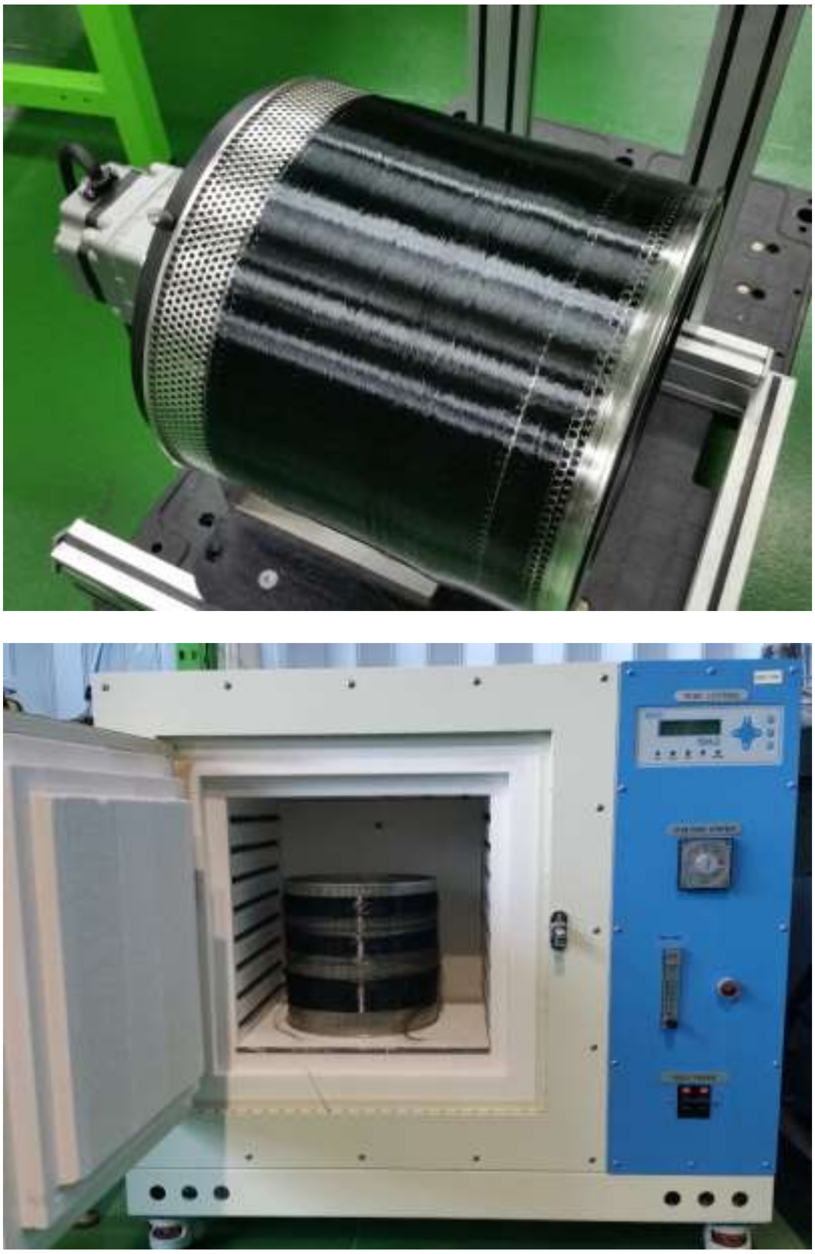

Figure S5. Image of a punched plate-based bobbin (top) and the oxidation setup (bottom).

Table S1. Oxygen and carbon content of IP- and MP-based pitch fibers after oxidation and carbonization for both batch- and screw-type extruder preparation

	Oxidation				Carbonization			
	Batch type		Screw type		Batch type		Screw type	
	O	C	O	C	O	C	O	C
IP0	7.2	82.8	-	-	7.9	91.3	-	-
IP1	17.2	72.7	19.3	74.1	6.6	92.4	5.1	93.5
IP4	20.4	71.5	21.4	72.9	4.7	95.1	3.0	95.8
IP8	25.1	66.2	25.2	69.8	4.7	94.8	1.4	96.2
IP12	27.2	65.4	26.4	68.7	5.2	92.6	1.8	96.0
IP16	-	-	27.7	67.9	-	-	4.3	94.2
MP0	6.8	86.3	-	-	7.7	91.0	-	-
MP1	11.2	85.3	7.1	87.5	6.2	91.7	5.7	91.9
MP4	13.8	79.1	15.7	79.3	4.3	94.7	3.3	95.8
MP8	17.2	75.6	19.1	76.1	1.3	97.4	1.4	97.8
MP12	16.3	75.1	19.7	76.9	3.4	95.8	3.6	95.1

* The calculated oxygen content: 100% - (sum of C, N, H, and S contents)

Table S2. Temperature profiles of the screw-type extruder and optimum process conditions

<Batch extruder>

**Conditions**	**IP**	**MP**
Temperature profiles	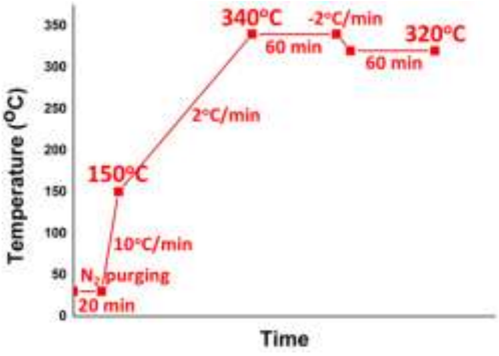	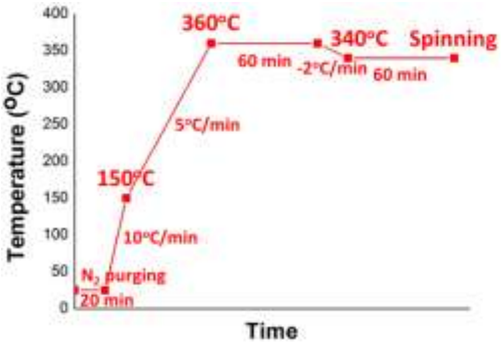
N_2 pressure (Kgf/cm^2)	1.0	1.0

<Screw extruder>

	← Feeder		**Barrel**		Diehead →		**Adap-ter**	**Gear pump**	**Nozzle**
	1	2	3	4	5	6			
IP	270	310	320	320	320	320	330	320	320
MP	290	330	340	340	340	340	350	340	350

	**RPM**		**Torque (N m)**	**Pressure (Kgf/cm^2)**	
	Screw	Gear pump		Diehead	Nozzle
IP	20	10	6~7	15~18	17~19
MP	20	10	4~5	16~20	25~27

Table S3. Oxygen mass content confirmed by X-ray photoelectron spectral analysis

OFs	IP1	IP4	IP8	IP12	MP1	MP4	MP8	MP12
Mass cont %	16.1	23.1	23.2	23.6	13.4	16.2	20.0	19.7

Table S4. Thermal and elemental analysis of pristine pitches and pelletized samples (pitch pellet)

	**IP**		**MP**	
Softening point (°C)	Powder	274.2	Powder	292.7
	Pellet	273.8	Pellet	291.3
DSC*	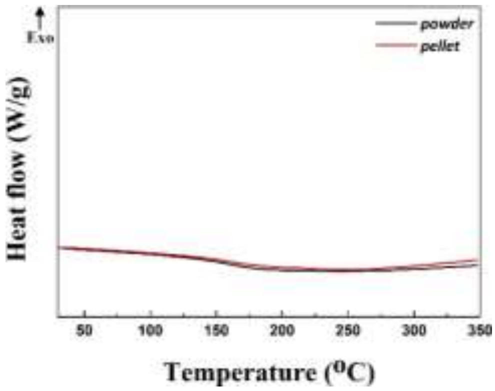		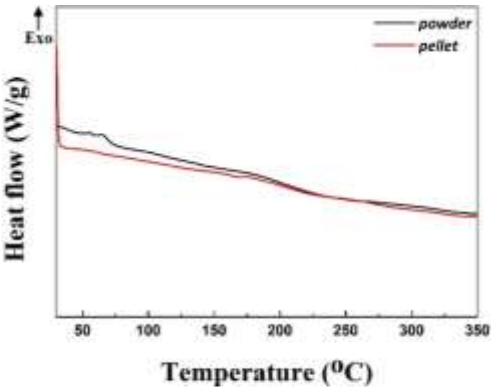	
TGA (N_2)**	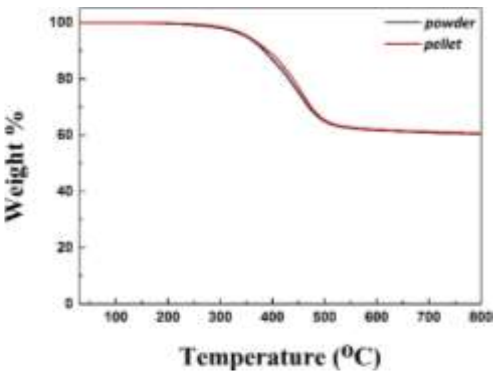		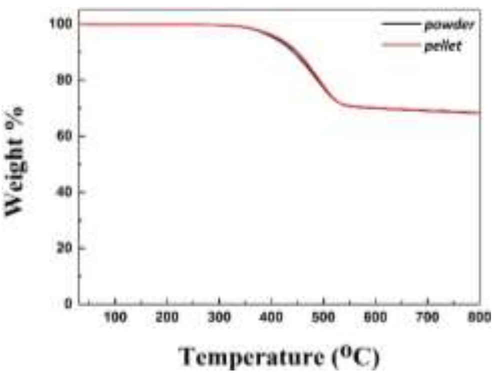	
TGA (O_2)**	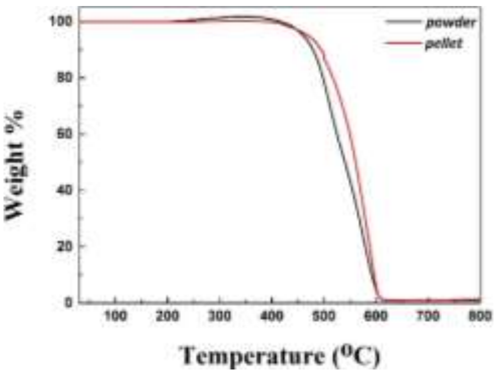		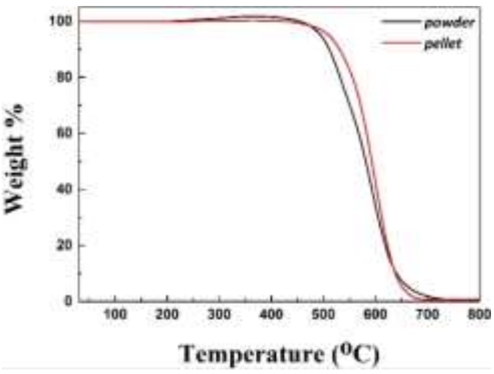	

EA		C	N	H	S	O		C	N	H	S	O
	Powder	92.2	0.0	5.2	0.0	2.6	Powder	94.6	0.0	4.3	0.4	0.7
	Pellet	93.1	0.0	4.6	0.0	2.3	Pellet	95.7	0.0	3.7	0.2	0.4

*Heating rate: 10°C/min

**Heating rate: 5 °C/min
